# Beneficial Role of Quercetin in Prevention and Treatment of Ischemic Stroke: Mechanisms of Action and Administration Strategies

**DOI:** 10.3390/molecules31173010

**Published:** 2026-08-27

**Authors:** Aleksandra Szychowska, Mikołaj Grabarczyk, Weronika Szczepańska, Ewa Smolińska, Andrzej Glabinski, Piotr Szpakowski

**Affiliations:** 1 The Nicolaus Copernicus Provincial Multispecialty Center for Oncology and Traumatology in Lodz, 93-513 Lodz, Poland; alexbielenin@gmail.com; 2 Department of Clinical Pharmacology, Medical University of Lodz, 90-153 Lodz, Poland; mikolaj.grabarczyk@stud.umed.lodz.pl; 3 J. Gromkowski Voivodship Specialist Hospital in Wroclaw, 51-149 Wroclaw, Poland; weronika.justynska@stud.umed.lodz.pl; 4 Central Teaching Hospital, Medical University of Lodz, 92-213 Lodz, Poland; ewa.smolinska@stud.umed.lodz.pl; 5 Department of Neurology and Stroke, Medical University of Lodz, Zeromskiego 113 Street, 90-549 Lodz, Poland; andrzej.glabinski@umed.lodz.pl

**Keywords:** polyphenols, quercetin, ischemic stroke, neurology

## Abstract

Polyphenols are a complex class of plant secondary metabolites that exert numerous beneficial effects on human health. The content of individual polyphenolic compounds varies considerably among plant species. Over the past few decades, polyphenols have attracted increasing interest because of their antioxidant and antimicrobial properties. They have also demonstrated beneficial effects in the prevention and treatment of various diseases, including cardiovascular and nervous system disorders, as well as different types of neoplasms. Because polyphenols have been shown to regulate plasma lipid levels and exhibit neuroprotective potential, they have gained attention as possible therapeutic and prophylactic agents for stroke, a neurological condition with a strong cardiovascular component. Quercetin is one of the most extensively studied plant-derived compounds in this context. A growing body of preclinical evidence suggests that it may contribute to stroke prevention and significantly support post-stroke recovery. This review compiles findings from in vitro studies, animal models, and clinical trials and presents the current state of knowledge regarding the biochemical and molecular mechanisms through which quercetin protects against cerebral ischemia.

## 1. Introduction

Among naturally occurring phytochemicals, or plant-derived compounds, polyphenols constitute one of the largest and most extensively studied groups because of their potential biomedical applications. To date, more than 8000 different compounds have been identified, and many more likely remain to be discovered. A defining structural feature of this group is the presence of an aromatic ring and one or more hydroxyl (-OH) groups [1,2]. Polyphenols are plant secondary metabolites that contribute to protection against harmful environmental factors and exhibit a wide range of biological activities including antioxidant, anti-inflammatory, antimicrobial, and neuroprotective effects [3,4,5,6]. Their antioxidant properties are largely associated with their ability to donate hydrogen atoms or electrons and thereby modulate reactions involving reactive species. Because polyphenols are widely present in commonly consumed plant-derived foods, their biological effects are of interest from both pharmacological and nutritional perspectives [3,4,5,6]. This review focuses on quercetin, a polyphenol belonging to the flavonol subclass. Its major dietary sources include red onions, apples, broccoli, and kale [7]. The presence of five hydroxyl substituents and a catechol group within the benzene ring contributes to quercetin’s antioxidant activity and its ability to interact with numerous biological targets [8]. These properties are particularly relevant to ischemic stroke, in which oxidative stress, neuroinflammation, apoptotic cell death, and disruption of the blood–brain barrier (BBB) contribute to secondary cerebral injury associated with ischemia and reperfusion [9,10,11]. Experimental studies using middle cerebral artery occlusion (MCAO) models have demonstrated that quercetin can reduce infarct volume, brain edema, and neurological deficits, while attenuating oxidative stress, apoptotic signaling, blood–brain barrier dysfunction, and inflammatory responses [9,10,11]. Epidemiological studies have also suggested that higher dietary intake of flavonols may be associated with a lower incidence of ischemic stroke [12]. Together, these findings provide a rationale for investigating quercetin as a potential multitarget neuroprotective compound in ischemic stroke. Despite its promising biological properties, the pharmaceutical application of quercetin is limited by an unfavorable pharmacokinetic profile. Quercetin is poorly soluble in water and exhibits limited intestinal absorption, while the absorbed fraction undergoes extensive first-pass metabolism, resulting mainly in methylated, glucuronidated, and sulfated derivatives [8]. These limitations may considerably reduce the concentration of biologically active quercetin reaching target tissues. Consequently, various strategies aimed at improving its bioavailability and tissue delivery have been investigated, including liposomes, exosomes, nanoformulations, and chemical modification with more soluble substituents [13,14]. Given its antioxidant, anti-inflammatory, anti-apoptotic, and other neuroprotective properties, quercetin represents a promising candidate for further investigation in the context of ischemic stroke. This review aims to evaluate the potential role of quercetin in the prevention and treatment of ischemic stroke, with particular emphasis on the molecular mechanisms underlying its neuroprotective effects, the pharmacokinetic limitations that may restrict its therapeutic efficacy, and strategies designed to improve its bioavailability. In addition, the review examines both direct and indirect evidence from clinical studies, including data specifically related to stroke incidence and outcomes as well as studies assessing cardiovascular risk factors and vascular effects relevant to stroke prevention. The novelty of this review lies in its integrated assessment of the entire translational pathway of quercetin in ischemic stroke—from molecular and preclinical evidence, through pharmacokinetic and delivery-related barriers, to the currently available human evidence. Particular attention is given to distinguishing direct clinical evidence related to stroke from indirect evidence, thereby enabling a more critical evaluation of the extent to which the promising experimental effects of quercetin can be translated into clinical practice.

## 2. Materials and Methods

### 2.1. Literature Search Strategy

A structured literature search was conducted to ensure a proper overview of the available literature. The literature search was performed independently by two researchers, exclusively using the PubMed database. The primary objective was to identify relevant studies investigating the pharmacological properties and therapeutic potential of quercetin, with a particular focus on its neuroprotective effects regarding ischemic stroke.

### 2.2. Search Terms and Strings

Researchers independently performed the literature search using the same specific keywords and Medical Subject Headings terms, combined using Boolean operators (AND, OR). The search strings included different combinations of the following terms: (“quercetin” OR “polyphenols”) AND (“stroke” OR “ischemic stroke” OR “cerebral ischemia” OR “MCAO” OR “middle cerebral artery occlusion”) AND (“neuroprotection” OR “oxidative stress” OR “inflammation” OR “apoptosis”).

### 2.3. Study Selection and Inclusion Criteria

After their independent literature searches, all retrieved records were merged into a single database and duplicate entries were subsequently removed. The remaining records were initially screened by title and abstract, after which potentially relevant articles underwent full-text review to assess their eligibility. Studies were included according to the following predefined criteria:

- In vitro and in vivo preclinical studies (e.g., MCAO animal models) exploring the mechanisms of action of quercetin;
- Clinical trials and randomized controlled trials evaluating the efficacy and bioavailability quercetin;
- Review articles and meta-analyses providing background context;
- Articles published in English up to the year 2026.

Studies not concerning the neuroprotective, anti-inflammatory, or antioxidant mechanisms of quercetin, as well as those not published in English, were excluded from this review. Any discrepancies regarding the final inclusion of a specific article were resolved through mutual discussion to reach a consensus.

## 3. Ischemic Stroke and the Potential Place for Polyphenols in Its Treatment

### 3.1. Epidemiology and Pathophysiology of Ischemic Stroke

Along with hemorrhagic stroke, ischemic stroke is one of the two main types of stroke and, according to epidemiological analyses, is the most common form of the disease. According to data from 2019, it accounted for approximately 62% of all stroke cases and was responsible for 3.3 million deaths worldwide [15]. The main pathological basis of this cerebrovascular disorder is the obstruction of blood flow in one or more cerebral arteries, leading to ischemia in the brain regions supplied by these vessels. This is predominantly caused by cardiovascular conditions such as atherosclerosis and thrombosis. In these conditions, embolic material may form within the vasculature, promoting excessive blood clotting in critical arteries or becoming detached. Such detached fragments may then travel through the vascular system and become lodged in smaller vessels, resulting in their occlusion [16,17]. The primary risk factors for ischemic stroke include metabolic and cardiovascular disorders such as hypertension, dyslipidemia, diabetes mellitus, and obesity. Individuals with conditions that increase the likelihood of thrombosis, such as atrial fibrillation, are also at higher risk [18].

### 3.2. Current Treatment Strategies and Their Limitations

Ischemic stroke is typically treated with intravenous thrombolysis using recombinant tissue-type plasminogen activator (r-TPA), namely alteplase, to dissolve the blood clot. However, this treatment is safe and effective only within a limited therapeutic time window. The European Stroke Society recommends that it should be administered only to patients who began to show stroke symptoms no more than 4.5 h earlier. Endovascular thrombectomy, that is, mechanical removal of the blood clot, may be performed instead in cases in which this time window has been exceeded or the patient does not qualify for thrombolytic treatment. However, this intervention should not be undertaken if more than 6 h have passed since the onset of stroke symptoms [16,17]. Even when successful reperfusion is achieved, patients may still suffer irreversible neuronal damage because the brain is deprived of oxygen and nutrients during vessel occlusion. This results in the loss of various neurological functions. Restoring lost brain function requires the rapid implementation of extensive rehabilitation. Even then, the pre-stroke condition may not be fully recoverable, leading to a marked reduction in independence and quality of life. As the incidence of stroke is projected to rise, the associated increase in mortality and disability-adjusted life years (DALYs) is becoming an increasingly important concern [15,19].

### 3.3. Polyphenols as Potential Therapeutic Agents in Ischemic Stroke

To expand the range of potential therapeutic and preventive options for patients with a history of stroke or those at increased risk, the search for new antithrombotic and neuroprotective agents has been ongoing for many years. To date, several compounds have been proposed as potential candidates; however, none have been approved or recommended in official guidelines issued by neurological and cardiological societies. Plant-derived polyphenols have also attracted interest in this field of research because of their anti-inflammatory and neuroprotective properties, their common presence in dietary sources, and their generally favorable safety and tolerability profiles. These compounds exert antioxidant effects by targeting multiple molecular pathways and influencing various aspects of neuronal metabolism. The beneficial outcomes observed under ischemic conditions include the restoration of mitochondrial function, stabilization of antioxidant enzyme activity, alleviation of endoplasmic reticulum stress, and inhibition of lipid peroxidation. Collectively, these effects may reduce stroke severity and enhance neuronal recovery, as demonstrated in various animal models of cerebral ischemia [20,21]. Studies examining the role of polyphenols in the central nervous system (CNS) indicate that they attenuate neuronal death by reducing oxidative stress and the overproduction of reactive oxygen species (ROS). This effect is associated with the upregulation of redox enzymes, including catalase (CAT) and superoxide dismutase (SOD). Moreover, because these phytochemicals stimulate nitric oxide synthase (NOS), they may contribute to the production of nitric oxide (NO) in endothelial cells, thereby inducing vasodilation and counteracting ischemia [20,21,22]. These vasodilatory properties may also be relevant to stroke prevention and the inhibition of atherogenesis. Additionally, polyphenols display immunomodulatory properties, influencing the inflammatory response of brain-resident mast cells and microglia, which may positively affect the post-stroke regeneration of cerebral tissue. Because polyphenols are also recognized for their beneficial effects on the cardiovascular system, they may contribute to stroke prevention through their antithrombotic and antiplatelet activities, which could reduce the likelihood of embolic material formation and thereby lower the risk of occlusion in major cerebral arteries [20,21,22]. Most of the currently available data on the role of polyphenols in the prevention or treatment of ischemic stroke come from preclinical studies based on cell cultures and animal models of cerebral ischemia. However, these findings demonstrate broad and potent biological activity of phytochemicals, particularly quercetin, in this context. Therefore, quercetin may be regarded as a serious and promising candidate for further evaluation in more advanced clinical trials, provided that the limitations associated with its generally poor pharmacokinetic profile can be overcome [20,21,22].

## 4. Measures to Increase Quercetin’s Bioavailability for the Central Nervous System

Limited water solubility and bioavailability of quercetin pose a significant challenge to clinical implementation. To overcome these limitations, new strategies enhancing the availability of quercetin in the body have been developed [8]. Among these are micellar drug-delivery systems and formulations, modification of quercetin with sugars, alternative routes of administration and others, which are discussed below.

### 4.1. Emulsification and Encapsulation

Among the most effective pharmacological strategies for increasing quercetin’s bioavailability, liposomes and solid dispersion formulations have emerged as particularly promising approaches. A study evaluating lecithin-based particles, known as phytosomes, as molecular carriers demonstrated that they increased the maximum plasma concentration (Cmax) achieved after oral administration of quercetin in human subjects by nearly 20-fold. This marked enhancement in polyphenol absorption was attributed to improved dispersion within the biological membranes of enterocytes [13]. Comparable findings were reported for a self-emulsifying micellar formulation composed predominantly of glycerin, purified water, medium-chain triglycerides, methylsulfonylmethane, and lecithin, enclosed in soft-gel capsules. In human participants, this formulation produced a 7- to 15-fold increase in the area under the plasma concentration–time curve (AUC) for quercetin compared to the reference formulation. The enhanced bioavailability was attributed to improved solubilization and intestinal absorption of quercetin, potentially accompanied by increased lymphatic transport and partial protection from rapid presystemic metabolism [14]. γ-Cyclodextrin, a cyclic oligosaccharide capable of forming inclusion complexes with poorly water-soluble compounds, has also been investigated as a strategy to improve quercetin bioavailability. Owing to its hydrophobic internal cavity and hydrophilic outer surface, γ-cyclodextrin can enhance the apparent solubility and gastrointestinal dispersibility of hydrophobic bioactive compounds. In humans, the oral administration of isoquercitrin (quercetin-3-O-glucoside) complexed with γ-cyclodextrin resulted in higher plasma concentrations of quercetin metabolites than the administration of isoquercitrin combined with dextrin, indicating enhanced systemic exposure [23].

### 4.2. Liposomes Enhanced with Molecular Anchors

Strategies based on liposomal formulations can be further optimized by modifying the surface layers of carrier particles with additional structures that act as molecular anchors for specific target tissues or improve solubility and pharmacokinetic properties. Transferrin and the peptide RVG29 are among the compounds with confirmed efficacy in this regard, as their incorporation into delivery systems enhances penetration of the BBB and improves brain-targeted delivery [24,25].

### 4.3. Modified Exosomes

Among the more recently developed classes of drug-delivery systems, exosomes have attracted considerable attention as promising carriers for targeted therapeutic delivery. As naturally derived extracellular vesicles composed of lipid bilayer membranes, they can interact efficiently with biological membranes and, importantly, may facilitate transport across the blood–brain barrier (BBB). In addition, their generally low immunogenicity and high biocompatibility contribute to their favorable safety profile [26]. In one particularly relevant approach, exosomes were engineered with a peptide selectively targeting CD86-positive microglia and loaded with quercetin. When administered in a murine model of ischemic stroke, this formulation reduced post-ischemic neuroinflammation and improved neurological recovery, highlighting the potential of exosome-based quercetin delivery for targeting inflammatory processes after cerebral ischemia [27]. Despite these advantages, the clinical translation of exosome-based delivery systems remains limited by several challenges, including relatively low drug-loading efficiency, difficulties in large-scale production and standardization, and potential variability in vesicle composition [26,27].

### 4.4. Glycosidation of Quercetin

An alternative strategy for improving quercetin bioavailability involves the modification of its chemical structure through glycosylation. Enzymatically modified isoquercetin consists primarily of quercetin-3-O-glucoside and its α-oligoglucosyl derivatives and has been reported to exhibit substantially greater bioavailability than either quercetin-3-O-glucoside or rutin [28]. Studies investigating enzymatically modified isoquercetin suggest that the introduction of α-glucosyl residues may enhance intestinal absorption, potentially by facilitating interactions with glucose-transport pathways and thereby providing an additional mechanism for translocation across the intestinal epithelium [29,30]. A related strategy has been explored using liposomal formulations in which quercetin was conjugated with glucose moieties. These modifications markedly enhanced the transport of quercetin-loaded liposomes across the BBB, apparently by exploiting glucose transporter 1 (GLUT1), which is highly expressed on brain endothelial cells. Importantly, glucose-functionalized liposomal systems exhibited greater BBB penetration than their unmodified counterparts, suggesting that combining nanocarrier-based delivery with transporter-mediated targeting may represent a particularly effective strategy for increasing the cerebral delivery of quercetin [31,32].

### 4.5. Alternative Routes of Administration

In addition to the use of different modifications and delivery systems, the bioavailability of quercetin and other polyphenols can also be increased by altering the route of administration. Parenteral delivery protects the administered compound from hepatic metabolism associated with the first-pass effect, thereby increasing the concentration of the biologically active form. Of particular importance for delivery to the central nervous system is the intranasal route of administration, as it provides access to brain tissue via the olfactory bulb. The efficiency of this pathway may be further enhanced by simultaneous use of formulations based on solid lipid nanoparticles or polymers such as poly(lactic-co-glycolic acid) (PLGA) or chitosan, owing to their mucoadhesive properties [33,34,35,36].

### 4.6. Advanced Physical Strategies: Magnetism and Ultrasound

The most technologically advanced delivery strategies include magnetically guided nanoparticles, particularly iron oxide-based systems. This approach enables drug-loaded carriers to be directed and concentrated within selected brain regions using an external magnetic field. However, its broader application remains limited by the need for specialized equipment and precise control of magnetic targeting parameters. A related physical strategy involves focused ultrasound (FUS), typically combined with intravenously administered microbubbles, to transiently and reversibly increase BBB permeability. Microbubble cavitation induced by ultrasound can facilitate the localized passage of circulating therapeutic agents into selected brain regions, providing a degree of spatial control that is difficult to achieve with systemic delivery alone. Nevertheless, precise regulation of the extent and duration of BBB opening is essential, as excessive ultrasound exposure or high microbubble doses may increase the risk of vascular damage and acute inflammatory responses [26].

### 4.7. Modulation of Liver Metabolism

**Figure 1:**
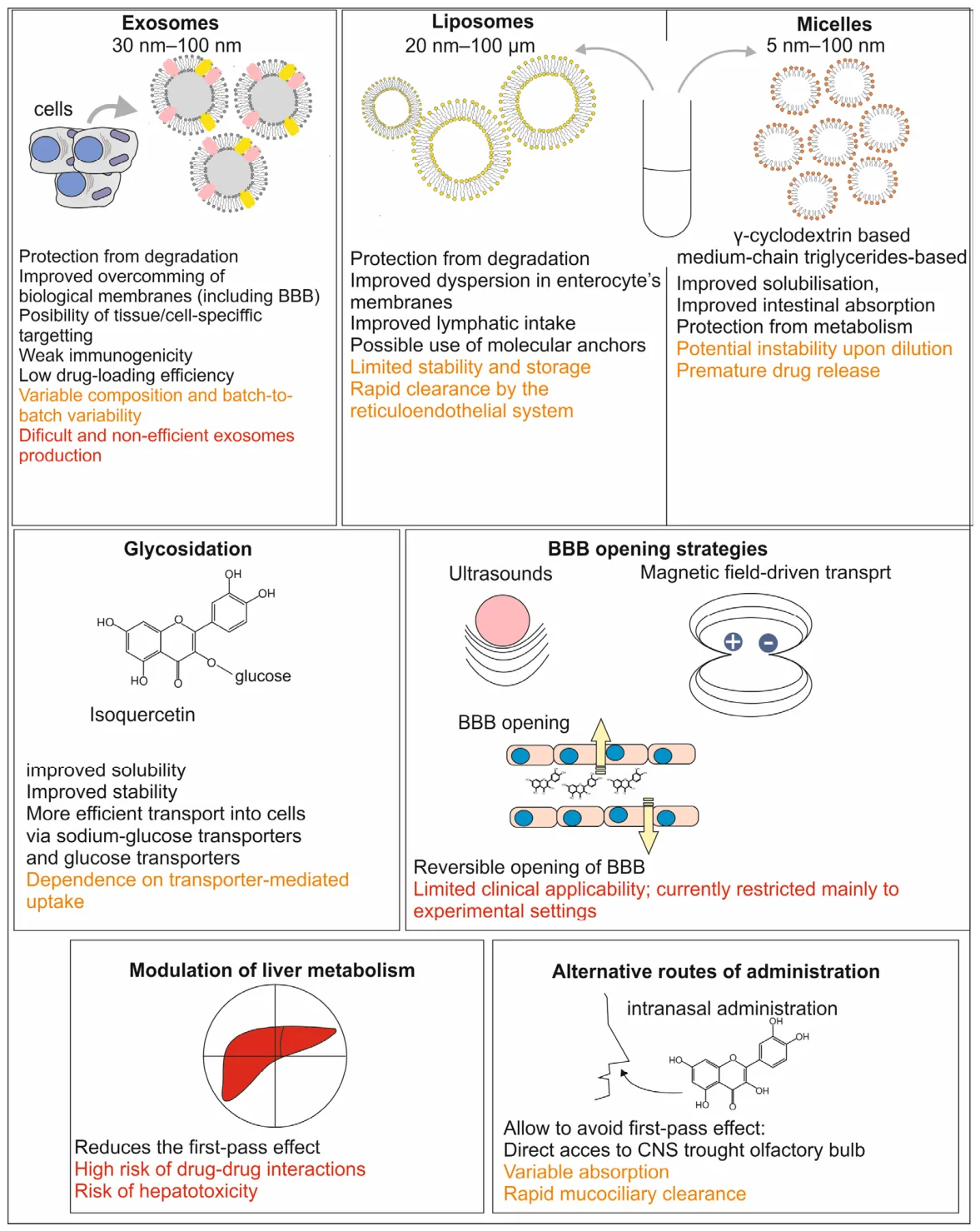
Strategies aimed at improving quercetin bioavailability and facilitating its delivery to the central nervous system. Red text highlights major translational, safety, or clinical applicability concerns, whereas orange text indicates additional technical or pharmacological limitations.

The significance of the first-pass effect may be reduced by co-administration of compounds that modulate hepatic metabolic pathways. Agents investigated in this context include piperine and sodium oleate, which may increase systemic exposure to quercetin without requiring chemical modification of the parent compound. However, modulation of hepatic enzymes and transporters may also affect the pharmacokinetics of concomitantly administered drugs. Consequently, compared to formulation-based strategies that primarily aim to improve quercetin solubility or absorption, this approach may carry a greater risk of clinically relevant drug–drug interactions [37]. Different strategies that allow improvement in quercetin’s bioavailability are summarized in Figure 1.

## 5. Quercetin’s Role in Treatment of Ischemic Stroke—Reports from In Vitro Studies

### 5.1. Effects of Quercetin on Endothelial Cells

Quercetin exhibits strong vasculoprotective and barrier-protective effects under ischemic and inflammatory conditions. In human aortic endothelial cells (HAECs), quercetin (5–20 μM) suppressed lipopolysaccharide (LPS)-induced expression of E-selectin and intercellular adhesion molecule 1 (ICAM-1), inhibited ROS generation, and activated the p38 mitogen-activated protein kinase/nuclear factor erythroid 2-related factor 2 (p38-MAPK/Nrf2) pathway, leading to the upregulation of the antioxidant enzymes heme oxygenase 1 (HO-1), NADPH quinone dehydrogenase 1 (NQO1), and glutamate-cysteine ligase (GCL). Because E-selectin and ICAM-1 are key mediators of leukocyte and thrombocyte adhesion, their inhibition suggests that quercetin may help prevent endothelial dysfunction, plaque progression, and thrombus formation [38]. Similar protective effects were observed in human brain microvascular endothelial cells (HBMECs) subjected to hypoxia and following reoxygenation. Quercetin improved cell viability and migration, enhanced angiogenesis, and reduced apoptosis and oxidative stress. These effects were associated with the activation of the Kelch-like ECH-associated protein 1/nuclear factor erythroid 2-related factor 2 (Keap1/Nrf2) pathway and the inhibition of activating transcription factor 6/78 kDa glucose-regulated protein (ATF6/GRP78)-mediated endoplasmic reticulum stress. Quercetin also restored mitochondrial membrane potential and upregulated the tight junction proteins claudin-5 and zonula occludens 1 (ZO-1), thereby protecting BBB integrity under ischemia-like conditions [39]. Beyond its effects on intracellular signaling, quercetin also modulates endothelial mitochondrial ion channels. In mitochondria isolated from human umbilical cord vascular endothelial cells, quercetin activated large-conductance mitochondrial calcium-activated potassium (mitoBKCa) channels by influencing their β3 subunit. Opening of mitoBKCa channels promotes mitochondrial depolarization and potassium influx into the matrix, thereby exerting an anti-apoptotic effect [40]. Consistent with this observation, quercetin dose-dependently increased mitochondrial membrane potential in HBMECs during hypoxia/reoxygenation, thus preventing apoptotic signaling and preserving endothelial mitochondrial function, a key determinant of BBB stability [39]. Recent studies indicate that quercetin enhances the protective effect of hydroxysafflor yellow A (HSYA) on the BBB by modulating Src/P-glycoprotein/matrix metalloproteinase 9 (Src/P-gp/MMP-9) signaling. Combined treatment preserved the integrity of human cerebral microvascular endothelial cell line D3 (hCMEC-D3) monolayers under oxygen-glucose deprivation (OGD), regulated the levels of Src, phosphorylated Src (p-Src), P-gp, and MMP-9, and improved the preservation of tight junctions. Molecular docking and molecular dynamics simulations confirmed stable, high-affinity binding of quercetin and HSYA to Src and P-gp, supporting their synergistic BBB-protective effects in ischemic stroke [41].

### 5.2. Effects of Quercetin on Macrophages

In THP-1 macrophages, quercetin (5–100 μM) exerts potent anti-inflammatory and antiatherogenic effects. It downregulated monocyte chemoattractant protein 1 (MCP-1) and ICAM-1, two key mediators of monocyte recruitment and adhesion, thereby inhibiting early stages of atherogenesis. Reduced MCP-1 expression limits monocyte migration into the subendothelial space, whereas decreased ICAM-1 expression weakens macrophage–endothelium interactions. Quercetin simultaneously enhanced cholesterol efflux, most likely through upregulation of liver X receptor alpha (LXR-α), cluster of differentiation 36 (CD36), and macrophage scavenger receptor 1 (MSR-1), consistent with the activation of the liver X receptor/retinoid X receptor (LXR/RXR) pathway. By preventing the transformation of macrophages into foam cells, quercetin may limit plaque development and vascular inflammation, both of which are closely associated with the risk of ischemic stroke [42].

### 5.3. Effects of Quercetin on Microglial Cells

The antineuroinflammatory properties of quercetin may help mitigate chronic inflammatory processes induced by microglial overactivation. Excessive microglial activation plays a crucial role in the neurodegeneration that accompanies the development of pathological conditions such as ischemic stroke. Quercetin strongly modulates microglial activation and polarization under ischemic stress. It promotes a shift from the pro-inflammatory M1 phenotype to the anti-inflammatory M2 phenotype through the regulation of the phosphoinositide 3-kinase/protein kinase B/nuclear factor kappa B (PI3K/Akt/NF-κB) and Toll-like receptor 4/myeloid differentiation primary response 88/nuclear factor kappa B (TLR4/MyD88/NF-κB) signaling pathways [11,43]. In primary microglia subjected to oxygen-glucose deprivation/reoxygenation (OGD/R), quercetin (5–80 μM) suppressed the mRNA expression of M1 surface markers, including CD86 and inducible nitric oxide synthase (iNOS), as well as pro-inflammatory cytokines such as tumor necrosis factor alpha (TNF-α), interleukin 1 beta (IL-1β), and interleukin 6 (IL-6). At the same time, it increased the mRNA expression of M2 surface markers, including CD206 and arginase 1 (Arg-1), together with anti-inflammatory cytokines such as interleukin 10 (IL-10) and transforming growth factor beta (TGF-β). Moreover, quercetin dose-dependently promoted phosphorylation of Akt and PI3K, while inhibiting the phosphorylation of the inhibitor of nuclear factor kappa B alpha (IκBα) and NF-κB. These mediators play important roles in the regulation of neuroinflammation, maintenance of BBB integrity, and modulation of M1 microglial activation [11].

In BV2 microglial cells, quercetin (10–50 μM) increased cell viability, reduced lactate dehydrogenase (LDH) release, and attenuated oxidative stress by decreasing ROS and malondialdehyde (MDA) levels while upregulating antioxidant enzymes, including superoxide dismutase 1 (SOD1), superoxide dismutase 2 (SOD2), glutathione peroxidase 1 (GPX1), and CAT. Quercetin also suppressed TLR4/MyD88/NF-κB signaling, leading to reduced production of IL-1β, IL-6, and TNF-α. By simultaneously inhibiting oxidative stress and pro-inflammatory cascades, quercetin prevents microglial overactivation, an important driver of neuronal degeneration in ischemic stroke [43].

### 5.4. Effects of Quercetin on Neuronal Cells

#### 5.4.1. Antioxidant and Anti-Apoptotic Effects

In primary rat hippocampal neurons subjected to oxygen-glucose deprivation/reoxygenation (OGD/R), isoquercetin markedly reduced oxidative stress by lowering ROS and MDA levels, restoring SOD and CAT activity, and preventing apoptosis through Nrf2 nuclear translocation. Activated Nrf2 suppressed the nicotinamide adenine dinucleotide phosphate oxidase 4/reactive oxygen species/nuclear factor kappa B (NOX4/ROS/NF-κB) signaling cascade, and Nrf2 knockdown abolished the neuroprotective effect of isoquercetin, highlighting the central role of this pathway [44]. Quercetin-induced p38-MAPK activation may also contribute to enhanced Nrf2 transcriptional activity [38].

#### 5.4.2. Anti-Ferroptotic Effects

Ferroptosis represents another important mechanism targeted by the neuroprotective activity of quercetin. It is a form of regulated, nonapoptotic cell death that is closely linked to stroke pathophysiology and is characterized by the iron-dependent accumulation of lipid peroxides, which exacerbates oxidative stress and ultimately leads to neuronal death. In HT-22 neurons exposed to hydrogen peroxide (H_2_O_2_) or erastin, quercetin increased cell viability, decreased MDA levels and intracellular iron accumulation, restored the expression of glutathione peroxidase 4 (GPX4) and ferritin heavy chain 1 (FTH1), and downregulated acyl-CoA synthetase long-chain family member 4 (ACSL4), thereby inhibiting ferroptotic injury. This antiferroptotic effect depended on Nrf2 activation, as the Nrf2 inhibitor ML385 reversed GPX4 restoration and abolished the protective effects of quercetin. Consistent with the in vitro findings, quercetin also reduced infarct volume and improved neurological outcomes in rats subjected to middle cerebral artery occlusion (MCAO), further supporting its ability to mitigate ferroptosis in vivo [45,46].

#### 5.4.3. Mitochondrial Protection

In primary cortical neurons exposed to OGD, quercetin (0.1–1 μM) enhanced mitochondrial respiration and cell survival, particularly when combined with epicatechin. Quercetin increased phosphorylation of cyclic adenosine monophosphate response element-binding protein (CREB) and upregulated the expression of CREB target genes involved in neuronal survival and mitochondrial biogenesis, namely B-cell lymphoma 2 (Bcl-2) and peroxisome proliferator-activated receptor gamma coactivator 1-alpha (PGC-1α), respectively. Quercetin also moderately increased intracellular Ca^2+^ levels, which elevated mitochondrial membrane potential and stimulated mitochondrial function, thereby protecting cells from apoptosis. By upregulating mitochondrial NADH dehydrogenase 2 (MT-ND2) and mitochondrial ATP synthase 6 (MT-ATP6), quercetin preserved spare respiratory capacity and provided marked neuroprotection following ischemic insult [47].

#### 5.4.4. Anti-Excitotoxic and Protease-Inhibitory Effects

μ-Calpain and hippocalcin are important molecular targets in hypoxia-induced neuronal injury driven by Ca^2+^ overload and oxidative stress. μ-Calpain is a Ca^2+^-activated cysteine protease that contributes to ischemic neurotoxicity by exacerbating intracellular Ca^2+^ influx and promoting proteolysis, whereas hippocalcin is a neuronal Ca^2+^ sensor that stabilizes intracellular Ca^2+^ levels and protects neurons from excitotoxic damage. Molecular docking data demonstrated that quercetin forms a stable inhibitory complex with μ-calpain, effectively suppressing Ca^2+^-dependent protease activity and limiting ischemia-related proteolysis [48]. In glutamate-exposed HT22 hippocampal neuronal cells, quercetin restored Ca^2+^ homeostasis, preserved hippocalcin expression, increased cell viability, reduced the expression of Bcl-2-associated X protein (Bax) and activation of caspase-3, and upregulated Bcl-2, thereby providing dose-dependent protection against glutamate-induced excitotoxicity [49].

#### 5.4.5. Ion-Channel Modulation

Another mechanism contributing to neuronal death during ischemic stroke is the activation of acid-sensing ion channels (ASICs), which promote apoptosis by increasing intracellular Ca^2+^ influx. In silico molecular docking studies demonstrated that quercetin has high binding affinity for ASICs, with reported binding energies of −7.35 kcal/mol and −9.3 kcal/mol [50,51]. By interacting with key residues within the catalytic region of acid-sensing ion channel subunit 1a (ASIC1a), quercetin modulates ASIC1a-mediated acidotoxicity and concurrently suppresses the activity of matrix metalloproteinase 2 (MMP-2) and MMP-9. These combined actions may provide dual protection against BBB disruption and acidotoxic Ca^2+^ overload [50,51]. Quercetin potently and reversibly inhibited homomeric rat ASIC1a, acid-sensing ion channel subunit 2a (ASIC2a), and acid-sensing ion channel subunit 3 (ASIC3), with a half-maximal inhibitory concentration (IC_50_) of approximately 2 μM, without affecting N-methyl-D-aspartate (NMDA), gamma-aminobutyric acid type A (GABAA), or purinergic receptor P2X4 channels. It also prevented low-pH-induced intracellular Ca^2+^ elevation and cell death in human embryonic kidney 293 (HEK-293) cells, which endogenously express ASIC1a and ASIC2a. Because ASIC3 is implicated in nociceptive signaling, its inhibition by quercetin may also contribute to pain relief and improved well-being in post-ischemic patients [52].

### 5.5. Effects of Quercetin Derivatives on Platelets and Vascular Cells

**Figure 2:**
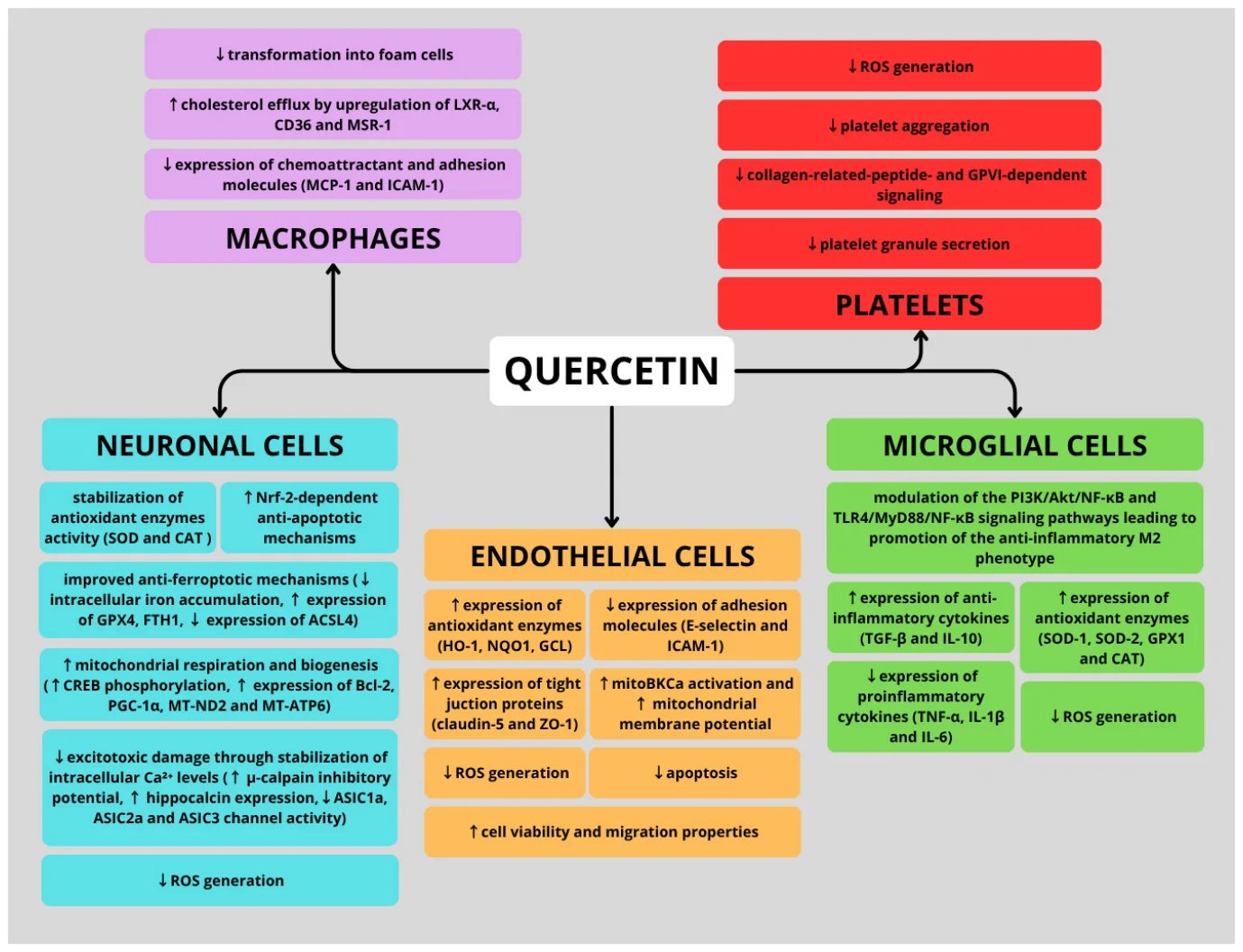
Protective effects triggered by quercetin in cell fractions associated with induction and progression of ischemic damage in central nervous system. ↑ means increase/upregulation; ↓ means decrease/downregulation. Figure created using Canva Free (Canva.com, accessed on 4 May 2026).

Beyond the nervous and glial systems, quercetin derivatives also exhibit significant antithrombotic and vasculoprotective effects. Quercitrin, a quercetin glycoside, inhibited collagen-related-peptide-induced platelet aggregation, platelet granule secretion, Ca^2+^ mobilization, and ROS generation by suppressing glycoprotein VI (GPVI)-dependent signaling and inhibiting the phosphorylation of key signaling proteins, including spleen tyrosine kinase (Syk), phospholipase C gamma 2 (PLCγ2), PI3K, Akt, and components of the TNF receptor-associated factor 4/p47^phox/hydrogen peroxide-inducible clone 5 (TRAF4/p47^phox/Hic-5) axis. Quercitrin also inhibited integrin αIIbβ3 outside-in signaling, thereby further reducing platelet activation. In vivo, oral quercitrin prevented FeCl_3_-induced arterial thrombosis under shear-flow conditions without prolonging bleeding time and reduced infarct volume in ischemia/reperfusion stroke models, underscoring its antithrombotic and neuroprotective potential [53]. The protective effects of quercetin, in various cell fractions important for induction and progression of ischemic damage in CNS, are summarized in Figure 2.

## 6. Quercetin’s Role in Treatment of Ischemic Stroke—Reports from Animal Models

### 6.1. Reports from Studies Where Quercetin Was Administered After the Onset of Cerebral Ischemia

One of the earliest reports on the neuroprotective effects of quercetin in animal models came from studies using the MCAO model. In these experiments, rodents received a quercetin–lecithin solution 30 min after the induction of cerebral ischemia. This intervention led to a significant reduction in brain lesion volume [54]. Subsequent studies using similar experimental designs not only confirmed these findings but also demonstrated several additional beneficial effects of this flavonol. Animals treated with quercetin showed improved post-ischemic tissue recovery, reduced cerebral edema, and less severe motor and cognitive impairment (Table 1). Biochemical analyses also revealed a smaller depletion of glutathione (GSH), one of the principal intracellular antioxidant molecules [41,43,44,55]. The observed reduction in edema may be attributable to decreased matrix metalloproteinase activity, particularly that of MMP-9, as excessive levels of this enzyme contribute to the disruption of BBB integrity [56]. Ghosh et al. and Ojo et al. reported that, under ischemic conditions, quercetin exerted protective effects on rat mitochondrial membranes. It reversed the loss of mitochondrial membrane microviscosity and alleviated the dysfunction of several mitochondrial enzymes and signaling components, including succinate dehydrogenase (SDH), Src kinase, and NADH-cytochrome c reductase. Ultimately, quercetin also promoted protective mitophagy, facilitating the removal of damaged mitochondria through mechanisms dependent on hypoxia-inducible factor 1 alpha (HIF-1α) and transcription factor EB (TFEB) [57]. This contributed to the preservation of efficient energy metabolism, which was reflected in reduced neuronal loss. The protective effect was particularly evident in the hippocampal cornu ammonis 1 and 3 (CA1 and CA3) regions [58,59]. In studies of chronic cerebral hypoperfusion using the bilateral common carotid artery occlusion (BCCAO) model, quercetin reduced ischemia-induced vacuolar changes in rat optic tracts, prevented excessive astroglial proliferation, and downregulated the oxidative stress marker nitrotyrosine [60]. The beneficial effects of quercetin also involve the modulation of apoptosis and DNA repair. Ahmad et al. demonstrated that quercetin counteracted the activity of poly(ADP-ribose) polymerase (PARP), an enzyme whose overactivation may deplete cellular adenosine triphosphate (ATP) reserves and thereby lead to cell death. Moreover, the analysis of cerebral tissue obtained from rats subjected to transient cerebral ischemia followed by reperfusion showed that this phytochemical decreased the expression of apoptosis-related factors, specifically caspase-3 and p53 [61]. Pyroptosis, a highly inflammatory form of programmed cell death, also plays a significant role in the progression of degenerative processes in the ischemic brain. It is driven predominantly by the NLR family pyrin domain containing 3 (NLRP3) inflammasome. Expression of this inflammasome is markedly increased in brain regions affected by stroke in rats; however, studies evaluating quercetin treatment have shown that this polyphenol can attenuate such upregulation and thereby counteract pyroptosis [62]. One of the key unresolved problems in stroke treatment is the oxidative damage that occurs after the restoration of blood flow. Although reperfusion is essential for salvaging ischemic neurons, the preceding transient deprivation of oxygen and nutrients alters cellular metabolism and significantly impairs antioxidant defenses. In this state, neurons become especially vulnerable to free radicals, which are inherent products of restored blood flow and aerobic metabolism. In studies of cerebral ischemia, quercetin was shown to support the activity of several rat enzymes involved in maintaining cytoplasmic redox balance, including SOD, CAT, and myeloperoxidase (MPO) (Table 1). This effect was particularly pronounced when quercetin administration was initiated before the onset of reperfusion [44,63,64]. Several authors attribute this antioxidant-promoting effect to the activation of the Nrf2/HO-1 pathway, which regulates the expression of cytoprotective proteins [62,65]. In addition to oxidative stress, excitotoxicity is another major factor contributing to damage and degeneration in ischemic regions of the CNS. ATP depletion impairs the function of ion pumps such as sodium/potassium adenosine triphosphatase (Na^+^/K^+^-ATPase), leading to uncontrolled depolarization and excessive release of neurotransmitters, particularly glutamate. Quercetin also appears to attenuate pathological alterations associated with this phenomenon. Research in rats using the 2-vessel occlusion (2VO) model showed that quercetin helped maintain Na^+^/K^+^-ATPase activity and preserved the function of glutamine synthetase (GS), an enzyme essential for protection against glutamate-induced neurotoxicity [59]. It is suspected that many of the neuroprotective effects exerted by quercetin are mediated through attenuation of abnormalities in cellular signaling pathways caused by the detrimental metabolic consequences of ischemia and reperfusion. Rats subjected to cerebral ischemia exhibit excessive dephosphorylation of extracellular signal-regulated kinase (ERK) and Akt, proteins that play key roles in cell survival. This alteration results from increased activity of tyrosine and serine/threonine phosphatases. Analyses performed in quercetin-treated animals revealed that these molecular disturbances were present to a significantly lesser extent, suggesting that quercetin may modulate the phosphorylation status of various enzymes and signaling proteins [66] (Table 1). This phenomenon was further investigated by Li et al., who observed that quercetin modulated the PI3K/Akt/NF-κB axis. By affecting this signaling pathway, quercetin influenced cerebral microglial polarization toward the M2 phenotype, which is considered particularly important for maintaining a cellular environment that supports neuroprotection and tissue regeneration [11]. In a study by Ulya et al., mice with stroke showed decreased expression of melanocortin 4 receptor (MC4R). Signaling pathways triggered by this membrane receptor can counteract prolonged or recurrent inflammatory and apoptotic responses, which are among the major factors worsening prognosis in animals and patients with ischemic stroke. Quercetin administration prevented downregulation of MC4R expression and alleviated the sensory and motor deficits observed in the examined mice [67]. The mammalian target of rapamycin (mTOR) axis plays an important role in the regulation of autophagy. It has been proposed that activation of this pathway protects the ischemic brain by limiting excessive degradation of cellular components. Alattar et al. found that quercetin treatment attenuated the reduction in phosphorylation of mTOR and p70S6 kinase in rodents subjected to MCAO. This was further reflected in a less pronounced decrease in the activity of downstream target proteins, including ribosomal protein S6, eukaryotic translation initiation factor 4E (eIF4E), and 4E-binding protein (4EBP) [62]. The results of studies assessing the effects of quercetin in animal models of cerebral ischemia in which treatment was initiated after the onset of ischemia are summarized in Table 1.

### 6.2. Reports from Studies Where Quercetin Was Administered Before the Onset of Cerebral Ischemia

As discussed in earlier sections of this review, the main intravascular pathology associated with an increased risk of ischemic stroke is atherosclerotic plaque formation. Most preventive strategies for the management of neurovascular diseases and the reduction in their incidence focus on preserving plaque stability and preventing plaque development. Quercetin may also have potential in this context as in a rabbit model of cholesterol-induced atherosclerosis, administration of this polyphenol inhibited the growth and progression of atherosclerotic plaques. This effect occurred without changes in serum lipid parameters and was associated with a reduction in proteasome activity in the trypsin-like (TL), chymotrypsin-like (CTL), and peptidyl–glutamyl peptide-hydrolyzing (PGPH) patterns to levels observed in healthy control animals. These effects were detected both in arterial tissue and in circulating leukocytes [68]. However, most studies have focused on quercetin as a neuroprotective pretreatment capable of attenuating ischemia-induced brain injury, rather than on its ability to prevent the cardiovascular events that lead to stroke (Table 2). Among the properties that make quercetin a promising agent for CNS protection are its stabilizing effects on biological membranes. Histological and molecular analyses of brain tissue obtained from quercetin-treated animals subjected to cerebral artery occlusion showed that this polyphenol protected the BBB against excessive increases in permeability and attenuated the loss of tight junction proteins, namely ZO-1, occludin, and claudin-5 [10]. Administration of quercetin encapsulated in nanocarrier particles 2 h before the onset of cerebral ischemia was associated with significantly lower production of conjugated dienes, major molecular markers of membrane-related oxidative stress. In the same study, such treatment also prevented ischemia/reperfusion-induced reductions in GSH levels and SOD and CAT activity. Moreover, it significantly reduced ROS generation [58]. Analyses performed by Shalavadi et al. revealed that pretreatment with quercetin also markedly attenuated the loss of total thiols, a group of compounds considered to be the main plasma-derived antioxidants [69]. Another important mechanism of protection against oxidative stress involves thioredoxin, a protein whose activity is highly dependent on the presence of thiol groups. Quercetin administration before the onset of cerebral ischemia was shown to mitigate the loss of thioredoxin in rat brain tissue and to enhance its inhibitory effect on apoptosis signal-regulating kinase 1 (ASK1), thereby exerting antiapoptotic activity [70]. In addition to confirming the beneficial effects of quercetin pretreatment on antioxidant enzymes and peptides, Viswanatha et al. demonstrated that such treatment alleviated the deterioration of motor and cognitive performance, thereby reducing the functional deficits caused by stroke [71]. This was also reflected at the histological level, as cerebral tissue samples obtained from treated animals showed fewer histopathological features of degeneration and injury, such as nuclear condensation, dendritic loss, and vacuole accumulation [9]. According to Shah et al., the neuroprotective effect associated with quercetin administration may result from its ability to modulate and stabilize the basal expression patterns of various proteins involved in cellular metabolism and differentiation. In rats subjected to cerebral artery occlusion, quercetin upregulated the expression of isocitrate dehydrogenase (IDH), adenosylhomocysteinase (AdoHcyase), pyruvate kinase, and ubiquitin carboxyl-terminal hydrolase L1 (UCH-L1). At the same time, it downregulated the expression of heat shock protein 60 (HSP60) and collapsin response mediator protein 2 (CRMP2). Overall, the resulting protein expression profile more closely resembled that observed in non-ischemic cells [72]. Park et al. found that one of the affected molecular targets was also protein phosphatase 2A (PP2A) subunit B. This subunit influences PP2A activity, which is responsible for regulating tau protein phosphorylation and the subsequent aggregation of cytoskeletal elements. Persistent impairment of PP2A activity is associated with the progression of neurodegenerative processes and can be detected in brain regions previously affected by ischemia [73]. Studies performed in rats subjected to stroke induced by the MCAO model revealed a less pronounced decrease in hippocalcin expression among animals treated with quercetin before the onset of ischemia. This may represent an important mechanism for limiting excitotoxic damage. The uncontrolled release of neurotransmitters, particularly glutamate, from ischemic neurons leads to excessive Ca^2+^ influx. Intracellular hippocalcin binds the resulting Ca^2+^ excess, thereby counteracting downstream effects mediated by Ca^2+^ as a second messenger [49]. Another signaling pathway positively influenced by quercetin pretreatment is the sirtuin 1/nuclear factor erythroid 2-related factor 2/heme oxygenase 1 (SIRT1/Nrf2/HO-1) axis. This pathway is associated with the initiation of antioxidant, anti-inflammatory, and antiapoptotic cellular responses, and several researchers consider it a crucial mediator of quercetin-induced protective mechanisms. Administration of this flavonol before ischemic stroke contributes to maintaining stable levels of the individual components of this pathway. This is reflected in a smaller increase in infarct volume, reduced ROS generation and BBB damage, and less severe neurological deficits [10]. The summarized results of studies evaluating the effects of quercetin in animal models of cerebral ischemia in which treatment was initiated before the onset of ischemia are presented in Table 2.

## 7. Quercetin’s Role in Treatment of Ischemic Stroke—Reports from Trials Involving Humans

In contrast to in vitro and animal studies, relatively few human clinical trials have directly investigated the role of quercetin in the treatment of ischemic stroke. Most human studies have focused on stroke risk factors or on mechanisms involved in stroke development and progression, such as increased platelet activation, endothelial dysfunction, elevated inflammatory markers, and ischemia–reperfusion injury. A cohort study by Knekt et al. investigated the relationship between quercetin intake and the subsequent incidence of cerebrovascular accident (CVA). The mean quercetin intake among individuals who developed CVA during the follow-up period was 3.57–4.07 mg/day. The study found no association between quercetin intake and a reduced incidence of thrombotic stroke or other cerebrovascular events in either men or women. Moreover, in individuals with diabetes, high quercetin intake was associated with an increased risk of thrombotic stroke. However, the authors emphasized that the absence of a protective association in their population may have resulted from methodological limitations, such as the very low quercetin dose and limited long-term reliability of exposure assessment [74]. A prospective cohort study of more than 55,000 Danish adults followed for approximately 21 years to examine the association between habitual flavonoid intake and the risk of ischemic stroke reached different conclusions. Higher or moderate consumption of flavonoid-rich foods, particularly sources of anthocyanins and flavanol oligomers and polymers, was associated with a lower incidence of ischemic stroke, suggesting a potential protective role of a flavonoid-rich diet. As an observational study, however, it demonstrates an association rather than a causal relationship [75]. Similar findings come from a cohort study including 2001 adults with chronic kidney disease from the U.S. NHANES database which investigated the association between dietary quercetin intake, stroke prevalence, and long-term survival. Higher quercetin intake was associated with a lower prevalence of stroke and reduced mortality. After adjustment for confounders, each 1 mg/day increase in quercetin intake was associated with approximately a 4% lower prevalence of stroke and a 2% lower risk of death, although the confidence intervals approached the null value, indicating borderline statistical significance [76]. Another cross-sectional analysis of 3806 older U.S. adults from the NHANES database investigated the association between dietary flavonol intake and stroke prevalence. Higher quercetin intake was associated with significantly lower odds of stroke, with participants in the highest intake category showing approximately 53% lower odds (OR = 0.472, 95% CI 0.245–0.907). However, a U-shaped relationship suggested that this association was not strictly linear across the full range of intake [77]. Beyond these epidemiological observations, human intervention studies provide additional, although indirect, evidence that quercetin may influence several mechanisms and modifiable risk factors involved in ischemic stroke development. These studies have primarily assessed platelet reactivity, endothelial and inflammatory biomarkers, blood pressure, oxidative stress, and lipid metabolism rather than stroke incidence or neurological outcomes. One potentially relevant mechanism is the modulation of platelet activation and thrombus formation. In the study by Hubbard et al., healthy participants received a single dose of 150 or 300 mg of quercetin-4′-O-β-D-glucoside. Platelet aggregation was significantly inhibited 30 and 120 min after ingestion of both doses. Quercetin also reduced collagen-stimulated tyrosine phosphorylation of platelet proteins, including Syk and phospholipase Cγ2, key components of the signaling pathway downstream of the platelet glycoprotein VI collagen receptor. These findings indicate that quercetin can acutely attenuate platelet activation and signaling in humans and therefore may have antithrombotic potential. However, the study assessed short-term changes in platelet function rather than thrombotic events, and its relevance to the prevention of ischemic stroke remains uncertain [78]. The effects of quercetin on endothelial dysfunction and systemic inflammation appear less consistent. Dower et al. conducted a randomized, double-blind, placebo-controlled crossover trial in 37 healthy prehypertensive men and women aged 40–80 years. Participants received quercetin-3-glucoside at 160 mg/day, epicatechin at 100 mg/day, or placebo for 4 weeks, with 4-week washout periods between interventions. Quercetin supplementation significantly reduced plasma concentrations of soluble E-selectin and IL-1β, whereas no significant changes were observed in most other biomarkers of endothelial dysfunction and inflammation, including soluble ICAM-1, soluble VCAM-1, MCP-1, CRP, serum amyloid A, TNF-α, IL-6, and IL-8 [79]. Moreover, in a double-blind randomized clinical trial conducted by Zahedi et al. in 72 women with type 2 diabetes, supplementation with 500 mg quercetin/day for 10 weeks did not significantly alter serum IL-6, TNF-α, or high-sensitivity CRP concentrations compared to placebo [80]. Similarly, studies involving overweight or obese adults with metabolic syndrome traits or individuals with prehypertension and stage 1 hypertension found little or no improvement in endothelial function or inflammatory biomarkers after supplementation with approximately 150–162 mg quercetin/day for 6 weeks [81,82]. Pfeuffer et al. likewise reported no improvement in endothelial function after 150 mg/day for 8 weeks and observed a moderate increase in TNF-α, suggesting that the anti-inflammatory effects of quercetin in humans are not consistent across populations and intervention protocols [83]. Among the cardiovascular risk factors relevant to stroke, the effect of quercetin on blood pressure appears somewhat more consistent, particularly in individuals with established hypertension. In a randomized, double-blind, placebo-controlled crossover study by Edwards et al., supplementation with 730 mg quercetin/day for 28 days significantly reduced systolic and diastolic blood pressure in participants with stage 1 hypertension, whereas no significant antihypertensive effect was observed in prehypertensive participants. Nevertheless, between-treatment differences were limited, and quercetin did not significantly alter markers of antioxidant capacity or oxidative stress, including the ferric reducing ability of plasma, plasma antioxidant reserve, and urinary 8-isoprostane F2α concentrations [84]. In the study by Egert et al., 150 mg quercetin/day reduced systolic blood pressure by 2.6 mmHg in the overall study population, by 2.9 mmHg in hypertensive participants, and by 3.7 mmHg in adults aged 25–50 years [81]. Brüll et al. similarly reported that 162 mg quercetin/day reduced mean 24 h systolic blood pressure by 3.6 mmHg, daytime systolic blood pressure by 4.6 mmHg, and nighttime systolic blood pressure by 6.6 mmHg in participants with stage 1 hypertension, together with reductions in mean arterial pressure. These effects were not observed in prehypertensive participants [82]. In individuals with type 2 diabetes, Zahedi et al. found that 500 mg quercetin/day for 10 weeks reduced both systolic and diastolic blood pressure within the intervention group, although only the reduction in systolic blood pressure was significantly greater than that observed with placebo (−8.8 ± 9.3 mmHg vs. −3.5 ± 11.7 mmHg) [80]. Pfeuffer et al. also reported a tendency toward lower fasting systolic blood pressure and a significant reduction in postprandial systolic blood pressure of approximately 5.7 mmHg after supplementation with 150 mg/day [83]. The mechanisms underlying these effects remain incompletely understood but may involve changes in vascular function, oxidative signaling, or modulation of the renin–angiotensin–aldosterone system [81,82]. In contrast, the effects of quercetin on lipid and metabolic risk factors have generally been modest or inconsistent. In most of the clinical studies described above, supplementation did not produce clinically relevant changes in triglycerides, total cholesterol, LDL cholesterol, VLDL cholesterol, HDL cholesterol, apolipoproteins, glucose, or insulin concentrations [80,81,82,83]. One study reported a significant reduction in plasma oxidized LDL concentrations following quercetin supplementation, suggesting a possible effect on lipoprotein oxidation rather than on the conventional lipid profile itself. Overall, however, the metabolic response to quercetin appears heterogeneous and may depend on baseline cardiovascular risk, dose, duration of supplementation, diet, lifestyle, and interindividual differences in quercetin metabolism [81]. Taken together, human intervention studies suggest that quercetin can influence selected biological processes that are relevant to ischemic stroke pathogenesis, particularly platelet activation and blood pressure, with less consistent effects on endothelial dysfunction, inflammation, oxidative stress, and lipid metabolism. These findings provide mechanistic support for a possible cerebrovascular protective effect but remain indirect, because the studies relied predominantly on surrogate cardiovascular and metabolic outcomes rather than incident ischemic stroke, recurrent vascular events, infarct characteristics, or post-stroke neurological and functional outcomes. Consequently, the available human evidence does not establish that quercetin supplementation prevents ischemic stroke or provides clinically meaningful neuroprotection. Further prospective clinical studies specifically designed to assess cerebrovascular endpoints are required to determine whether the protective effects observed in experimental models translate into benefits in humans.

## 8. Conclusions

Quercetin emerges from the available literature as a promising phytochemical with multifaceted biological activities relevant to both the prevention and treatment of ischemic stroke. The evidence reviewed in this paper indicates that its effects extend beyond direct antioxidant activity and involve the regulation of multiple simple antioxidant action and involve broad regulation of molecular and cellular processes central to ischemic brain injury. In experimental models, quercetin and its derivatives protect endothelial cells, preserved blood–brain barrier integrity, attenuate oxidative stress, reduce neuroinflammation, and limit neuronal death. These benefits have been associated with modulation of several interconnected signaling pathways, including PI3K/Akt/NF-κB, TLR4/MyD88/NF-κB, Keap1/Nrf2, and SIRT1/Nrf2/HO-1, as well as with improved mitochondrial function, suppression of ferroptosis, mitigation of excitotoxicity, and inhibition of apoptotic and pyroptotic signaling. In addition, quercetin demonstrated antithrombotic, antiatherogenic, and vasculoprotective properties, which may be particularly important in view of the strong cardiovascular component of ischemic stroke. The preclinical findings are supported by a wide range of in vitro and in vivo studies showing that quercetin may reduce infarct volume, cerebral edema, BBB disruption, and neurological deficits, while improving post-ischemic tissue recovery and functional outcomes. Importantly, beneficial effects were observed both when quercetin was administered after the onset of ischemia and when used as pretreatment, suggesting possible value in both therapeutic and preventive settings. Nevertheless, these findings remain predominantly preclinical, and their translation into clinically meaningful cerebrovascular protection has yet to be demonstrated. Human evidence is considerably more limited and heterogeneous. Epidemiological studies have produced mixed findings, with some observational analyses suggesting associations between higher dietary quercetin or flavonoid intake and a lower prevalence or incidence of stroke, whereas others have failed to demonstrate a protective association. Human intervention studies provide additional mechanistic evidence that quercetin may favorably affect selected processes relevant to stroke pathogenesis. The most consistent signals include acute inhibition of platelet activation and modest reductions in blood pressure, particularly in individuals with established hypertension, whereas effects on endothelial dysfunction, systemic inflammation, oxidative stress, lipid metabolism, and other metabolic parameters have been less consistent. Importantly, these studies have predominantly evaluated surrogate cardiovascular and metabolic endpoints rather than stroke incidence, recurrent cerebrovascular events, infarct characteristics, or neurological and functional outcomes. Thus, no direct clinical evidence currently demonstrates that quercetin supplementation prevents ischemic stroke or improves outcomes after acute cerebral ischemia. At the same time, the reviewed data clearly show that the clinical translation of quercetin remains limited by its poor pharmacokinetic profile, including low water solubility, inefficient intestinal absorption, and extensive first-pass metabolism. Considerable progress has been made in addressing these issues through liposomes, nanoformulations, exosomes, glycosylated derivatives, and alternative routes of administration, especially intranasal delivery, yet these strategies still require further validation. More advanced approaches, including magnetically guided nanoparticles and focused ultrasound-mediated transient BBB opening, may further improve brain-targeted delivery, although their technological complexity and safety requirements currently limit their clinical applicability. Strategies based on modulation of hepatic metabolism may also increase systemic exposure but require particular caution because of the potential for clinically relevant drug–drug interactions. Despite promising improvements in quercetin bioavailability achieved with several of these approaches, their ability to enhance therapeutic efficacy in patients with ischemic stroke remains to be established. Overall, the available evidence supports a strong mechanistic rationale for the further investigation of quercetin as a potential cerebrovascular protective and neuroprotective agent, but also reveals a substantial gap between experimental efficacy and clinical validation. Quercetin should therefore currently be regarded as a promising but investigational candidate rather than an established intervention for stroke prevention or treatment. Future research should prioritize well-designed randomized clinical trials with clinically relevant cerebrovascular endpoints, while simultaneously defining effective dosing regimens, pharmacokinetic targets, optimal brain-delivery strategies, and long-term safety. Such studies will be essential to determine whether the broad protective effects consistently observed in experimental models can ultimately be translated into meaningful benefits for individuals at risk of ischemic stroke or patients recovering from cerebral ischemia.

## Figures and Tables

**Table 1:** Summarized results from in vivo studies investigating the use of quercetin as treatment in ischemic stroke.

Dosage	Animal Species	Cerebral Ischemia Model	Way of Administration	Time Period	Results	Ref.
50 mg/kg	Male Sprague–Dawley rats	MCAO	Intraperitoneal injections, made every day, for 3 days after the onset of ischemia period	After 1.5 h of ischemia period, arteries were unblocked, starting a 72 h long reperfusion period after which the animals were sacrificed	↓ brain infarction volume ↓ neurological deficits ↓ M1 microglia/macrophage presence ↑ M2 microglia/macrophage presence ↓ CD86, TNF-α, IL-1β, and IL-6 expression levels ↑ CD206, Arg-1, IL-10, and TGF-β expression levels ↓ loss of p-PI3K and p-Akt expression ↓ rise of p-IκBα and p-NF-κB expression	[11]
10 mg/kg	C57BL/6 male mice	MCAO	Not specified	After 1.5 h of ischemia period, arteries were unblocked. The exact time of the animals being sacrificed was not specified.	↓ brain infarction volume ↓ neurological deficits ↓ BBB leakage	[41]
50 mg/kg	7 days old neonatal mice	HIBI	Intraperitoneal injections, made at various time points (0, 24 and 48 h) after the induction of ischemia period.	Mice were sacrificed 28 days after the induction of ischemia period	↓ brain infarction volume ↓ motor and cognitive deficits	[43]
30 mg/kg (in form of lecithin-based solution)	Male Sprague-Dawley rats	MCAO	Single intraperitoneal injection (30 min after the onset of ischemia period)	Rats were sacrificed 24 h after induction of ischemia period	↓brain infarction volume	[54]
30 mg/kg (in form of lecithin-based solution)	Male Sprague-Dawley rats	MCAO	Single intraperitoneal injection (30 min after onset of ischemia period)	Rats were sacrificed 24 h after induction of ischemia period	↓ brain infarction volume ↓ cerebral edema ↓ neuronal degeneration in the ipsilateral cortex and striatum ↓ neurological deficits ↓ loss of GSH levels	[55]
2.7 mg/kg (in form of PLGA-based nanoparticles)	Male Sprague Dawley rats	BCCAO	Oral administration (1 dose per day for 3 days after the onset of ischemia period)	Depending on the subgroup, the ischemia period lasted for 30 min, 24 h or 72 h after which the rats’ arteries were unblocked. All animals were sacrificed 72 h after the induction of ischemia period	↓ iNOS expression in hippocampal region ↑ mitochondrial membrane protection ↓ neuronal loss in hippocampal region ↓ caspase-3 activity in hippocampal region	[58]
20 mg/kg	Male Wistar rats	2VO	Intraperitoneal injections, made at the start of the reperfusion period and again after 2 h.	After 30 min of ischemia period, arteries were unblocked, starting a 24 h long reperfusion period after which the animals were sacrificed	↓ motor and cognitive deficits ↓ MDA and NO levels ↓ striatal and hippocampal XO and MPO activity ↓ loss of hippocampal GS, Na^+^ K^+^ATPase, AChe and TH activity ↓ loss of GPx, SOD and CAT activity ↓ hippocampal LDH and MAO activity ↓ loss of mitochondrial SDH, SCR and NADH-cytochrome c reductase activity	[59]
200 mg/kg	Male Wistar rats	BCCAO	Intraperitoneal injections (1 dose every 4 days)	Rats were sacrificed 8 weeks after the ligation	↓ vacuoles area in optic tract ↓ nitrotyrosine levels ↓ astroglial proliferation	[60]
30 mg/kg	Male Wistar rats	MCAO	Intraperitoneal injections made at various time points (1 h before and 0, 24, 48 and 72 h after the ischemia period onset)	After 2 h of ischemia period the rats’ arteries were unblocked, starting a 72 h long reperfusion period after which the animals were sacrificed	↓ brain infarction volume ↓ motor coordination deficits ↓ TBARS levels ↑ GSH activity ↓ loss of GPx, GR, GST, SOD, Na^+^ K^+^-ATPase and CAT activity ↓ upregulation of caspase-3 and PARP ↓ expression of p53	[61]
10 mg/kg	Male rats	MCAO	Intraperitoneal injections, made every day, for 3 days after the onset of ischemia period	After 1.5 h of ischemia period, arteries were unblocked, starting a 72 h long reperfusion period after which the animals were sacrificed	↓ degeneration of neurons ↓ DNA fragmentation ↓ loss of mTOR and P70S6 phosphorylated forms ↓ loss of S6, eIF4E, FKHR and 4EBP proteins ↑ Nrf2 and HO-1 expression ↓ NLRP3 levels ↓ LDH activity	[62]
50 mg/kg	Sprague Dawley rats	BCCAO	Single intraperitoneal injection (10 min before the onset of ischemia period)	After 30 min of ischemia period the rats’ arteries were unblocked, starting a 4 h long reperfusion period after which the animals were sacrificed	↓ brain infarction volume ↓ lipid peroxidation ↓ loss of SOD and CAT activity ↓ MPO activity	[63]
10 mg/kg	Swiss Albino mice	BCCAO	Oral administration (1 dose per day for 7 days after the onset of ischemia period)	After 15 min of ischemia period, arteries were unblocked, starting a 7 days long reperfusion period after which the animals were sacrificed	↓ brain infarction volume ↓ motor and cognitive deficits ↓ TBARS levels ↓ loss of GSH level ↓ loss of SOD activity	[64]
3.4 mg/mL (applied in GAP43-targetted exosomes)	Sprague-Dawley rats	MCAO	Single intravenous injection made at the start of the reperfusion period	After 2 h of ischemia period, arteries were unblocked, starting a 24 h long reperfusion period after which the animals were sacrificed	↓ brain infarction volume ↓ neurological deficits ↓ ROS generation ↑ Nrf2, NQO-1, and HO-1 levels ↓ loss of NeuN-positive cells ↓ neuronal malformations observed in histopathological analyses	[65]
25 mg/kg	Male Sprague-Dawley rats	BCCAO	Oral administration (1 dose per day for 21 days after the onset of ischemia period, with first dose applied before the occlusion)	After 1.5 h of ischemia period, arteries were unblocked, starting a 21 days long reperfusion period after which the animals were sacrificed	↓ brain infarction volume ↓ motor deficits ↑ BBB integrity ↓ caspase-3 activity ↓ MDA levels ↓ TNF-α and IL-1β expression ↓ tyrosine and serine/threonine phosphatase activity in ipsilateral cortical tissues	[66]
200 mg/kg	Swiss albino mice	occlusion of the left common carotid artery.	Intraperitoneal injections, made every day, for 7 days after the onset of ischemia period	After 2 h of ischemia period, arteries were unblocked, starting a 14 days long reperfusion period after which the animals were sacrificed	↓ motor and sensory deficits ↓ loss of MC4R expression	[67]

Abbreviations: 2VO, 2 vessel occlusion; 4EBP, 4E binding protein; AChe, acetylcholinesterase; Arg-1, arginase 1; BBB, blood–brain barrier; BCCAO, bilateral common carotid artery occlusion; CAT, catalase; CD86, cluster of differentiation 86; eIF4E, eukaryotic translation initiation factor 4E; FKHR, forkhead in rhabdomyosarcoma; GAP43, growth associated protein 43; GPx, glutathione peroxidase; GR, glutathione reductase; GS, glutamine synthetase; GSH, glutathione; GST, glutathione S-tranferase; HIBI, hypoxic–ischemic brain injury; HO-1, heme oxygenase 1; IL-10, interleukin 10; IL-1β, interleukin 1β; iNOS, inducible nitric oxide synthetase; LDH, lactate dehydrogenase; MAO, monoamine oxidase; MC4R, melacortin 4 receptor; MCAO, middle cerebral artery occlusion; MDA, malonyldialdehyde; MPO, myeloperoxidase; mTOR, mammalian target of rapamycin; NADH, nicotinamide adenine dinucleotide reduced form; NLRP3, NLR family pyrin domain containing 3; NO, nitric oxide; NQO, NADPH quinone oxidoreductase; Nrf2, nuclear factor erythroid 2-related factor 2; PARP, poly-ADP-ribose polymerase; p-IκBα, phosphorylated nuclear factor of kappa light polypeptide gene enhancer in B-cells inhibitor alpha; PLGA, polylactic-co-glycolic acid; p-NF-κB, phosphorylated nuclear factor kappa B; p-PI3K, phosphorylated phosphoinositide 3-kinase; SDH, succinate dehydrogenase; SOD, superoxide dismutase; TBARS, thiobarbituric acid reactive substances; TGF-β, transforming growth factor β; TH, tyrosine hydroxylase; TNF-α, tumor necrosis factor α; XO, xanthine oxidase. Notation: ↑ means increase/upregulation; ↓ means decrease/downregulation.

**Table 2:** Summarized results from in vivo studies investigating the use of quercetin as pretreatment in cerebral ischemia.

Dosage	Animal Species	Cerebral Ischemia Model	Way of Administration	Time Period	Results	Ref.
30 mg/kg	Male Sprague-Dawley rats	MCAO	Single intraperitoneal injection (1 h before the onset of ischemia period)	Rats were sacrificed 24 h after the induction of ischemia period	↓ neurological deficits ↓ brain infarction volume ↓ cerebral oedema ↓ loss of dendrites and whole neurons ↓ PARP and caspase-3 expression	[9]
50 mg/kg	Male Wistar rats	MCAO	Intraperitoneal injections, made every 2 days, for 6 days preceding the onset of ischemia period	After 1.5 h of ischemia period, arteries were unblocked, starting a 24 h long reperfusion period after which the animals were sacrificed	↓ brain infarction volume ↓ neurological deficits ↓ neuronal damage ↓ ROS generation ↑ protection against BBB permeability rise ↓ loss of ZO-1, occludin and claudin-5 ↓ loss of Sirt1 and Nrf2 expression	[10]
2.7 mg/kg (in form of PLGA-based nanoparticles)	Male Sprague Dawley rats	BCCAO	Oral administration (1 dose 2 h before the onset of ischemia period)	Depending on the subgroup, the ischemia period lasted for 30 min, 24 h or 72 h after which the rats’ arteries were unblocked. All animals were sacrificed 72 h after the induction of ischemia period	↓ conjugated diene generation ↓ ROS generation	[58]
25 mg/kg	Sprague-Dawley rats	BCCAO	Oral administration (1 dose per day for 10 days preceding the onset of ischemia period)	After 30 min of ischemia period, arteries were unblocked, starting a 24 h long reperfusion period after which the animals were sacrificed	↓ brain infarction volume ↑ BBB integrity ↓ LPO activity ↓ loss of SOD, CAT activity ↓ loss of GSH an total thiols levels	[69]
10 mg/kg	Male Sprague-Dawley rats	MCAO	Single intraperitoneal injection (30 min before the onset of ischemia period)	Rats were sacrificed 24 h after the induction of ischemia period	↓ brain infarction volume ↓ cerebral oedema ↓ motor and sensory deficits ↓ loss of thioredoxin expression ↓ impairment of ASK1 and thioredoxin interaction	[70]
20 mg/kg	Male Wistar rats	BCCAO	Intraperitoneal injections, made every day, for 7 days preceding the onset of ischemia period	After 1 h of ischemia period the rats’ arteries were unblocked, starting a 24 h long reperfusion period after which the animals were sacrificed	↓ brain infarction volume ↓ cerebral oedema ↓ motor and memory deficits ↓ lipid peroxidation ↓ loss of SOD and CAT activity ↓ loss of GSH levels	[71]
10 mg/kg	Male Sprague-Dawley rats	MCAO	Single intraperitoneal injection (30 min before the onset of ischemia period)	Rats were sacrificed 24 h after the induction of ischemia period	↓ neurological deficits ↓ brain infarction volume ↓ loss of PP2A subunit B expression in cerebral cortex	[73]

Abbreviations: ASK1, apoptosis signal-regulating kinase 1; BBB, blood–brain barrier; BCCAO, bilateral common carotid artery occlusion; CAT, catalase; GSH, glutathione; LPO, lipid peroxidation; MCAO, middle cerebral artery occlusion; Nrf2, nuclear factor erythroid 2-related factor 2; PARP, poly(ADP-ribose) polymerase; PLGA, poly(lactic-co-glycolic acid); PP2A, protein phosphatase 2A; ROS, reactive oxygen species; Sirt1, sirtuin 1; SOD, superoxide dismutase; ZO-1, zonula occludens-1; Notation: ↑ means increase/upregulation; ↓ means decrease/downregulation.

## Data Availability

The data used to support the findings of this study are included in this article.
